# Atlanta Medical College—Our Journal, Etc.

**Published:** 1861-08

**Authors:** 


### EDITORIAL AND MISCELLANEOUS.

### Atlanta Medical College, Our Journal, etc.

Much of the heretofore latent talent and energy of the South will now become thoroughly aroused, as sequences of secession and the pending war; nor have we ever believed that the ultimate effects of our conflict with the North will be disastrous to the people of the Confederacy. On the contrary, thus far our arms have been signally victorious, and that too against a most fearful odds, and the *signs of the times* warrant the conclusion that, at no distant day, we will have consummated a complete and final geographical, political and commercial severance from that fanaticism and agrarianism, which for many long years, has been but a disgrace and a curse to the *once* United States. Soon we expect to be a homogeneous people; soon we will be blessed with a holier ministry and a purer church-membership; soon we will erect an uncontaminated standard of literature, commensurate with the majesty of Southern mind, and more congenial to Southern taste; soon will we have a government administered under the guidance of Astrea, by patriot statesmen, and where the inalienable principle of *equal rights* will be sacredly observed; and with these inestimable blessings, may we not also reasonably expect that the sciences of Law and Medicine will share proportionably in the benefits of this radical change. The talents, learning and labors of our own great Chapman, and Horner, and Gibson, our Drake and Meigs, and Dickson and Sims, have mainly written the reputation of the Medical Colleges of the North, and Southern patronage has mainly thrown broad-cast the luxuries of affluence into the laps of her Faculties. Too long have we bowed (and we blush to confess it), in almost truckling subserviency to a boasted supremacy, neither legitimate or necessary. Too long have the talent of the South, and the products of her soil contributed to enrich the ungrateful and mercenary North. Henceforth let us not only plant, but cultivate in a more congenial soil; let us live and labor at home, and *for home;* and let us harvest and garner at home. Occupying as we do a lattitude, of all others, the most renowned for the production and development of human greatness, is not hope full of future fruition? Situated as is our own Institution in the very heart and centre of the Southern Confederacy, and at the identical locality marked out by the prophetic genius of the immortal Calhoun, as destined to become the Capital of a great Southern Confederacy, and born, as we were, almost simultaneous with the very throes which gave us our present national existence, with a Board of Trustees, a Faculty, and hosts of Alumni, all emphatically Southern; and foremost as we have been *ab initio*, politically and professionally, in the glorious cause of secession, it is but natural that our sentiments and sympathies are what they are; and we have no apology to make for an unreserved expression of the same, even though it be upon the pages of a Medical Journal.

Our Class of 1861, though not as large as usual, numbers more than was anticipated in consideration of the belligerent agitations of our country; for it is but too true that war and its dire consequences never fail to affect all the relations of life, with both the victorious and vanquished, and extends its ravages into all the departments of our interests and pursuits. We suppose, however, that we have not suffered more than our winter schools have, or are likely to; and even if we have, the sacrifice is a free-will offering upon Freedom's altar. The cause of secession, of the South, is our cause. With it we are willing to rise, and, if necessary, to fall. Her destiny shall be ours. Three of our colleagues are now connected with our invincible army on the Potomac, all battling professionally, and otherwise, in behalf of the glorious principles of right and honor. And, side by side with them are scores of our gallant Alumni—God bless them—winning golden honors for their profession, their country, and themselves. Well done noble sons of Æsculapius! Show your faith by your works! Prove to the enemy that you can *kill* or *cure* as occasion may demand.

But while we are proud to herald the strength of our representatives upon the tented field, and under the banner of Mars, we are by no means unmindful of that peaceful flag which we have unfurled in the sacred cause of Therapia—the cause of science and humanity. Those of us, not at the seat of war, are here, and, with might and main endeavoring to enlarge our facilities for the next "Summer Course," determined, whether there be *peace* or *war*, that all things shall be in readiness for our Class of 1862. And from abundant indications, "should grim visaged war have smoothed his wrinkled front," ere another May shall come, we expect to see our spacious halls crowded as heretofore, and the Summer School of the South again moving onward and upward to a still wider sphere of usefulness and distinction.

Among our absentees at the seat of war is the Editor of our *Journal*, but no interruption will occur in the publication and circulation of this periodical; and we are authorized to say to its patrons that arrangements have been made by which its pages will be materially enriched by more than the ordinary amount of original matter. But let not this good news relax the energies or diminish the efforts of that valuable aid which we have received from our numerous and able contributors heretofore. For now and henceforth, more than ever, must the South, in medicine, as in all things else, rely upon her own resources. And it is fortunate for us that our strength is at last about to be tested. Our population, our soil and climate, our diseases and their causes, have their peculiarities, and especially, into these peculiarities, let our investigations extend. Upon us, as a profession, this responsibility exclusively rests. Then, let us meet it in the spirit of true philanthropy; let us shake off the lethargy which has too long encumbered our strength, and convince the world that Southrons can wield the sceptre of science as well as that of war. Let us not fall behind our countrymen in the pulpit and the forum, or those at the bar or in the field.

We have no disposition to encourage sectional prejudices, no desire to foster the wormwood of national hatred, and if the recent infamous assault made by Drs. Conant Foster and John McNulty, upon Dr. F. Gaillard Thomas, in the New York Academy of Medicine, is to be taken as a specimen of Northern medical magnanimity and fraternization, we feel that, so far as the profession there is concerned, we have nothing to envy—much to commisserate, and something to despise. We are glad to learn, however, that that body, by a unanimous vote, administered to those two would-be *prominents* a well deserved rebuke. To Dr. Thomas we have only a word to say, and that is "come out from among them"—"neither cast ye your pearls before swine, lest they trample them under their feet, and turn *again* and rend you." And if you are guilty of the alleged offense, that is, of being a "*secessionist*," rejoice that you are, by birth, a Carolinian, and accounted worthy of the stripes.

